# Short-term local predictions of COVID-19 in the United Kingdom using dynamic supervised machine learning algorithms

**DOI:** 10.1038/s43856-022-00184-7

**Published:** 2022-09-24

**Authors:** Xin Wang, Yijia Dong, William David Thompson, Harish Nair, You Li

**Affiliations:** 1 School of Public Health, Nanjing Medical University, Nanjing, China; 2 Centre for Global Health, The University of Edinburgh, Edinburgh, UK; 3 Edinburgh Medical School, College of Medicine and Veterinary Medicine, The University of Edinburgh, Edinburgh, UK; 4 Division of Rheumatology, Orthopaedics and Dermatology, School of Medicine, University of Nottingham, Nottingham, UK

**Keywords:** Epidemiology, Epidemiology, Disease prevention

## Abstract

**Background:**

Short-term prediction of COVID-19 epidemics is crucial to decision making. We aimed to develop supervised machine-learning algorithms on multiple digital metrics including symptom search trends, population mobility, and vaccination coverage to predict local-level COVID-19 growth rates in the UK.

**Methods:**

Using dynamic supervised machine-learning algorithms based on log-linear regression, we explored optimal models for 1-week, 2-week, and 3-week ahead prediction of COVID-19 growth rate at lower tier local authority level over time. Model performance was assessed by calculating mean squared error (MSE) of prospective prediction, and naïve model and fixed-predictors model were used as reference models. We assessed real-time model performance for eight five-weeks-apart checkpoints between 1st March and 14th November 2021. We developed an online application (COVIDPredLTLA) that visualised the real-time predictions for the present week, and the next one and two weeks.

**Results:**

Here we show that the median MSEs of the optimal models for 1-week, 2-week, and 3-week ahead prediction are 0.12 (IQR: 0.08–0.22), 0.29 (0.19–0.38), and 0.37 (0.25–0.47), respectively. Compared with naïve models, the optimal models maintain increased accuracy (reducing MSE by a range of 21–35%), including May–June 2021 when the delta variant spread across the UK. Compared with the fixed-predictors model, the advantage of dynamic models is observed after several iterations of update.

**Conclusions:**

With flexible data-driven predictors selection process, our dynamic modelling framework shows promises in predicting short-term changes in COVID-19 cases. The online application (COVIDPredLTLA) could assist decision-making for control measures and planning of healthcare capacity in future epidemic growths.

## Plain language summary

Understanding how COVID-19 case numbers will change in the short term in local contexts is helpful for planning public health measures. Here, using population data on Google searches on COVID-19 symptoms, mobility (i.e., movement of people between locations), and vaccination coverage, we develop statistical methods that forecast changes in COVID-19 case numbers for the next one to three weeks in different local authorities in the UK. We further develop an online application that provides visualisations of these forecasts. We show that our programmes achieve better predictive accuracy compared with two existing models. This highlights the promise of our methods in forecasting future local COVID-19 outbreaks.

## Abstract (web summary)

Wang et al. predict local COVID-19 incidence one to three weeks ahead based on symptom search trends, population mobility, and vaccination coverage. Their dynamic model is more accurate than naïve and fixed-predictor models.

## Introduction

The COVID-19 pandemic has caused a substantial health and economic burden in the UK and globally. In the UK, SARS-CoV-2 has caused about 22 million cases, 960 thousand hospital admissions, and 201 thousand deaths as of 12th August 2022^1^. Given the continuous spread of SARS-CoV-2, short-term predictions are important to assist effective decision making for control measures and planning of healthcare capacity^2–4^.

Population aggregated digital big data based on personal digital devices and applications have been widely used to predict COVID-19 epidemics as they capture well the changes in population behaviours without disclosing personal information, in a near-real-time manner. Population-level mobility and internet searches are two of the most important digital data metrics that are commonly used in modelling infectious disease outbreaks. Population-level mobility data are publicly accessible from several sources, including Google COVID-19 Community Mobility Reports^5^, Apple COVID-19 Mobility Trends^6^, and Facebook Data for Good^7^, and have been increasingly used to understand changes in public physical contacts during the COVID-19 pandemic^8,9^. Between the sources, data are collected and provided differently. Our earlier work found that mobility at different types of locations are associated with varying degrees of changes in transmission of SARS-CoV-2, and visits to retail and recreation areas, workplaces, and transit stations are key drivers of COVID-19 epidemic in the UK^10^. Moreover, online search queries on Google have been used to detect and predict influenza and other infectious diseases before the COVID-19 pandemic^11–13^. The search trends of infection-related symptoms reflect infected people searching their symptoms in real time, thus can also be used as an early warning of the confirmed cases that are subject to the testing capacity and delays^14,15^.

Integrating the two metrics above that capture different types of population behaviours may improve the prediction from the perspective of COVID-19 early warning^3,16^. Kogan and colleagues^16^ developed an early warning approach to monitor COVID-19 activity with multiple digital databases among three states of the US, which showed that the predictions of COVID-19 cases and deaths were improved by integrating multiple digital traces. However, no dynamic models were applied in that study that allowed for data-driven adjustment over time (e.g., a fixed set of COVID-19 symptom search terms was used), and the predictions were made for March–September 2020, before the emergence of variants of concern and the mass roll-out of the COVID-19 vaccination. The Zoe COVID study^17^ found that there was addition of new symptoms over time, e.g., after the vaccination or potentially associated with the emergence of new variants. Moreover, the COVID-19 vaccination coverage may be also relevant to the prediction given that the mass rollout of COVID-19 vaccines could have reduced COVID-19 transmission^18^. As the COVID-19 situation changes rapidly, it is essential for COVID-19 prediction modelling studies to develop flexible algorithms adaptive to the most up-to-date data.

In this study, we predicted the short-term changes in the COVID-19 epidemics at finer geographical scale in the UK, through a dynamic supervised machine learning algorithm that could reflect the best real-time prediction informed by data on internet searches on COVID-19 symptoms, mobility, and vaccination coverage. The programmes achieved better predictive accuracy compared with two reference models, showing promises in forecasting future local COVID-19 outbreaks. Furthermore, we developed a publicly accessible web application to present the predictions.

## Methods

### Overview

Our primary objective was to develop data-driven machine-learning models for 1-, 2- and 3-week ahead predictions of growth rates in the COVID-19 cases (defined as 1-, 2- and 3-week growth rate, respectively) at lower-tier local authority (LTLA) level in the UK. In the UK, COVID-19 cases are reported by publication date (i.e., the date when the case was registered on the reporting system) and by the date of collection of specimen. Therefore, there were six prediction targets in our study, 1-, 2- and 3-week growth rates by publication date and those by the date of collection of specimen (Table 1). We focused on prediction by publication date in the main models, considering that the delayed reporting for COVID-19 cases by the collection date of specimen could affect real-time assessment of model performance (i.e., the prediction would be biased downwards due to delayed reporting).

**Table 1:** Prediction targets.

Outcome	Mathematic formula
*Y*1*p*_*t*_: 1-week-ahead change in COVID-19 cases (as 1-week growth rate) compared with week *t*, by publication date^a^	${\log }\frac{{{{{{\rm{Case}}}}}}{{P}}_{t+1}}{{{{{{\rm{Case}}}}}}{{P}}_{t}}$
*Y*2*p*_*t*_: 2-week-ahead change in COVID-19 cases (as 2-week growth rate) compared with week *t*, by publication date	${\log }\frac{{{{{{\rm{Case}}}}}}{{P}}_{t+2}}{{{{{{\rm{Case}}}}}}{{P}}_{t}}$
*Y*3*p*_*t*_: 3-week-ahead change in COVID-19 cases (as 3-week growth rate) compared with week *t*, by publication date	${\log }\frac{{{{{{\rm{Case}}}}}}{{P}}_{t+3}}{{{{{{\rm{Case}}}}}}{{P}}_{t}}$
*Y*1*s*_*t*_: 1-week-ahead change in COVID-19 cases (as 1-week growth rate) compared with week *t*, by collection date of specimen^b^	${\log }\frac{{{{{{\rm{Case}}}}}}{{S}}_{t+1}}{{{{{{\rm{Case}}}}}}{{S}}_{t}}$
*Y*2*s*_*t*_: 2-week-ahead change in COVID-19 cases (as 2-week growth rate) compared with week *t*, by collection date of specimen	${\log }\frac{{{{{{\rm{Case}}}}}}{{S}}_{t+2}}{{{{{{\rm{Case}}}}}}{{S}}_{t}}$
*Y*3*s*_*t*_: 3-week-ahead change in COVID-19 cases (as 3-week growth rate) compared with week *t*, by collection date of specimen	${\log }\frac{{{{{{\rm{Case}}}}}}{{S}}_{t+3}}{{{{{{\rm{Case}}}}}}{{S}}_{t}}$

Case*P*_*t*_ – number of COVID-19 cases by publication date at week *t*; Case*S*_*t*_ – number of COVID-19 cases by collection date of specimen at week *t*.

^a^Publication date refers to the date when the case was registered on the reporting system.

^b^Collection date of specimen refers to the date when the respiratory specimen was taken for testing.

### Data sources

We analysed the Google Search Trends symptoms dataset^5^, the Google Community Mobility Reports^19,20^, COVID-19 vaccination coverage and the number of confirmed COVID-19 cases for the UK^1^. These data were formatted and aggregated from daily to weekly level where needed, and then linked by week and LTLA. We considered only the time series from 1st June 2020 (defined as week 1) for modelling, given that case reporting was relatively consistent and reliable at LTLA level after 1st June 2020. The modelling work initially began on 15th May 2021 and was continuously updated using the latest available data since then; when models were fit, only the versions of the data that were available in real time were used. In this study, we used 14th November 2021 as the time cut-off for reporting (i.e., data between 1st June 2020 and 14th November 2021 were included for modelling) although our model continues to update regularly.

The Google symptom search trends show the relative popularity of symptoms in searches within a geographical area over time^21^. We used the percentage change in the symptom searches for each week during the pandemic compared to the pre-pandemic period (the three-year average for the same week during 2017–2019). We considered 173 symptoms for which the search trends had a high-level completeness in the analyses. These search trends were provided by upper-tier local authorities, and were extrapolated to each LTLA. The Google mobility dataset records daily population mobility relative to a baseline level for six specific areas, namely workplaces, residential areas, parks, retail and recreational areas, grocery and pharmacy, and transit stations^22^. The weekly averages of each of the six mobility metrics for each LTLA were the model inputs. The mobility in LTLAs of Hackney and City of London were averaged, given that they were grouped into one LTLA in other datasets. Cornwall and Isles of Scilly were combined likewise. The COVID-19 vaccination coverage dataset records the cumulative percentage of population vaccinated with the first dose of vaccine and that for the second dose on each day. Before the start of the vaccination rollout (7th December 2020 for first dose and 28th December 2020 for second dose), the coverage was deemed to be zero. We used the weekly maximum cumulative percentage of people vaccinated for the first dose and second dose for each LTLA in our models. Missing values on symptom search trends, mobility, and vaccination coverage were imputed using linear interpolation for each LTLA^23^. Thirteen LTLAs were excluded as data were insufficient to allow for linear interpolation.

### Models

#### Algorithm for model selection

We developed a dynamic supervised machine learning algorithm based on log-linear regression. The algorithm could allow the optimal prediction models to vary over time given the best available data to date, and therefore reflected the best real-time prediction given all available data.

**Fig. 1:**
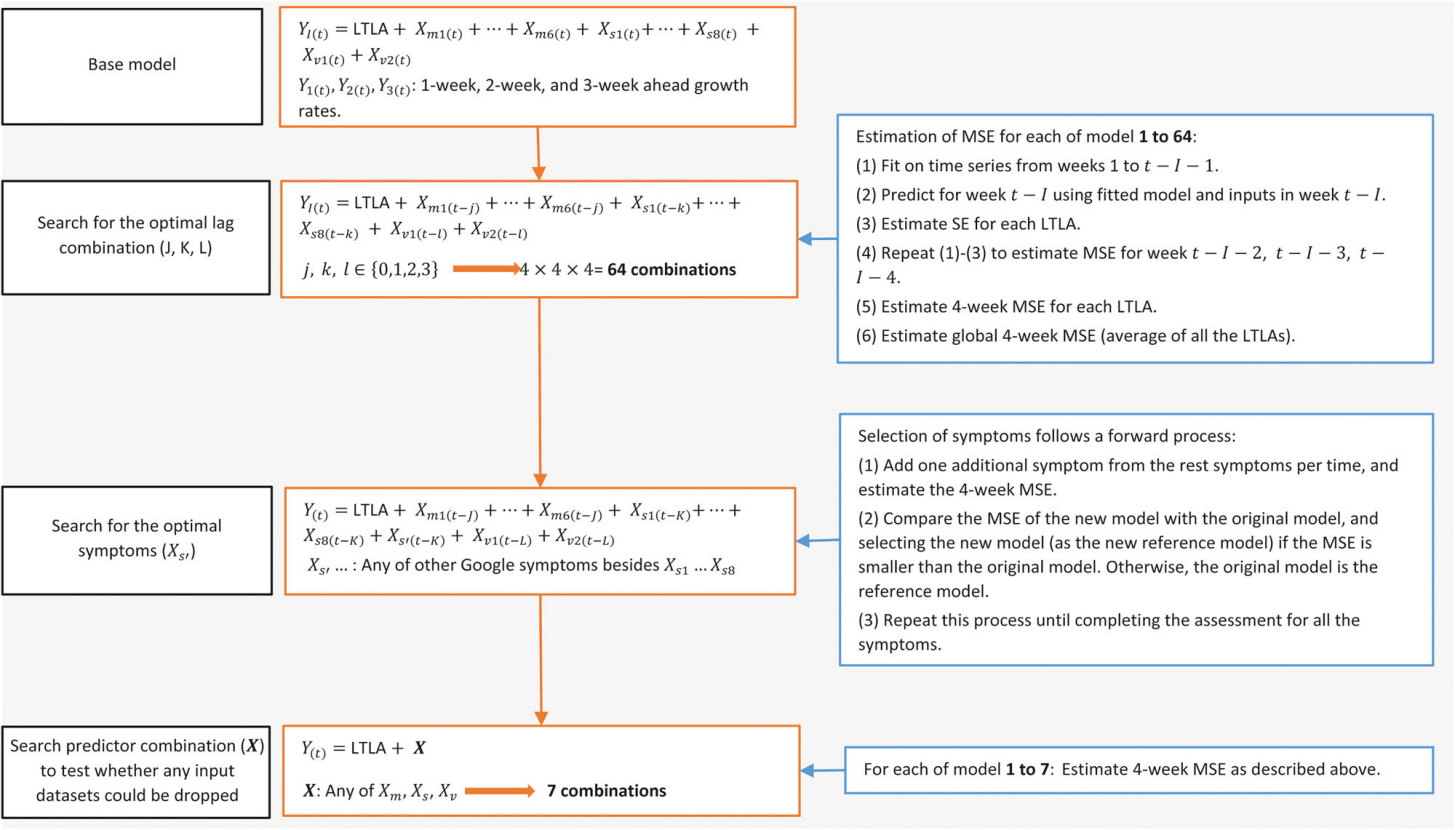
Schematic figure showing model selection and assessment. SE squared error, MSE mean squared error. In each of the assessment steps, the optimal model had the smallest MSE. *X*_*m*1(*t*)_ to *X*_*m*6(*t*)_: mobility metrics at six locations. *X*_*s*1(*t*)_ *to X*_*s*8(*t*)_: search metrics of the eight base symptoms. *X*_*v*1(*t*)_ *and X*_*v*2(*t*)_: COVID-19 vaccination coverage for the first and second dose. Details are in Supplementary Method.

Figure 1 shows the iteration of model selection and assessment. We started with a baseline model^24^ that included LTLA (as dummy variables), the six Google mobility metrics, vaccination coverage for the first and second doses, and eight base symptoms from the Google symptom search trends, including cough, fever, fatigue, diarrhoea, vomiting, shortness of breath, confusion, and chest pain, which were most relevant to COVID-19 symptoms based on existing evidence^25^. Dysgeusia and anosmia as the two other main symptoms of COVID-19^26^ were not included as base symptoms because Google symptom search data on the two symptoms were only sufficient to allow for modelling in about 56% of the LTLAs (the two symptoms were included as base symptoms in the sensitivity analysis described below). We then selected and assessed the optimal lag combination^15,27,28^ between each predictor and growth rate. Next, starting from the eight base symptoms, we applied a forward data-driven method for including additional symptoms in the model. This would allow the inclusion of other symptoms that could improve model predictability. Lastly, we assessed the different predictor combinations (Fig. 1; Supplementary Methods and Supplementary Table 1).

At each of the steps, model performance was assessed through calculating an average mean squared error (MSE) of the predictions over the previous four weeks, i.e., 4-week MSE, with the MSE for each week being evaluated separately by fitting the same candidate model (Fig. 1 and Supplementary Methods). The calculated 4-week MSE reflected the average predictability of candidate models over the previous four weeks (referred to as retrospective 4-week MSE). Models with minimum 4-week MSE were considered for inclusion in each step. Separate model selection processes were conducted for each of the prediction targets.

In addition, we considered naïve models as alternative model candidates for selection; naïve models (which assumed no changes in the growth rate) carried forward the last available observation for each of the outcomes as the prediction. Similar to the full models (i.e., models with predictors), we considered a time lag between zero and three weeks, and used the 4-week MSE for naïve models (Supplementary Table 2).

#### Prospective evaluation of model predictability

After selection of the optimal model based on the retrospective 4-week MSE, we proceeded to evaluating model predictability prospectively by calculating the prediction errors for forecasts of growth rates in the following 1–3 weeks (for the three prediction timeframes), referred to as prospective MSE (Supplementary Methods and Supplementary Table 3). As the optimal prediction models changed over time under our modelling framework, we selected a priori eight checkpoints that were five weeks apart for assessing model predictability (we did not assess every week due to the considerable computational time required): year 1/week 40 (the week of 1st March 2021), 1/45 (5th April), 1/50 (10th May), 2/3 (14th June), 2/8 (19th July), 2/13 (30th August), 2/18 (4th October) and 2/23 (14th November). For each checkpoint, we presented the composition of the optimal models as well as the corresponding prospective MSE.

Two reference models were used to help evaluate our dynamic optimal models. We considered naïve models (with optimal time lag based on 4-week retrospective MSE) as the first reference model, to understand how much the models driven by covariates could outperform models that assume status quo. As the second reference model, to further demonstrate the advantages of our dynamic model selection approach over the conventional model with a fixed list of predictors, we used the optimal model for the first checkpoint (i.e., year 1/week 40) and fixed its covariates (referred to as fixed-predictors model); then we compared its prospective MSEs for the next seven checkpoints (i.e., year 1/week 45 onwards), allowing the model coefficients to vary.

#### Sensitivity analyses

As sensitivity analysis, the base symptoms were expanded to further include dysgeusia and anosmia, as well as headache, nasal congestion, and sore throat that have been recently reported as common symptoms of COVID-19^17^ to assess how the predictive accuracy was influenced.

### Web application

We developed a web application COVIDPredLTLA using R ShinyApp, presenting our best prediction results at local level of the UK given all available data to date. COVIDPredLTLA (https://leoly2017.github.io/COVIDPredLTLA/), officially launched on 1st December 2021, uses real-time data from the above sources and currently updates twice per week. The application presents the predicted percentage changes (and uncertainties where applicable) in the COVID-19 cases in the present week (nowcasts) and the one and two weeks ahead (forecasts) compared with the previous week, using the optimal models (which technically could be naïve models or any of the full models), by two forms (publication date and the collection date of specimen) for each LTLA.

Analyses were done with R software (version 4.1.1). We followed the STROBE guidelines for the reporting of observational studies as well as the EPIFORGE guidelines for the reporting of epidemic forecasting and prediction research. All the data included in the analyses were population-aggregated data available in the public domain and therefore, ethical approval was not required.

### Reporting summary

Further information on research design is available in the Nature Research Reporting Summary linked to this article.

## Results

### Summary of input time series

**Fig. 2:**
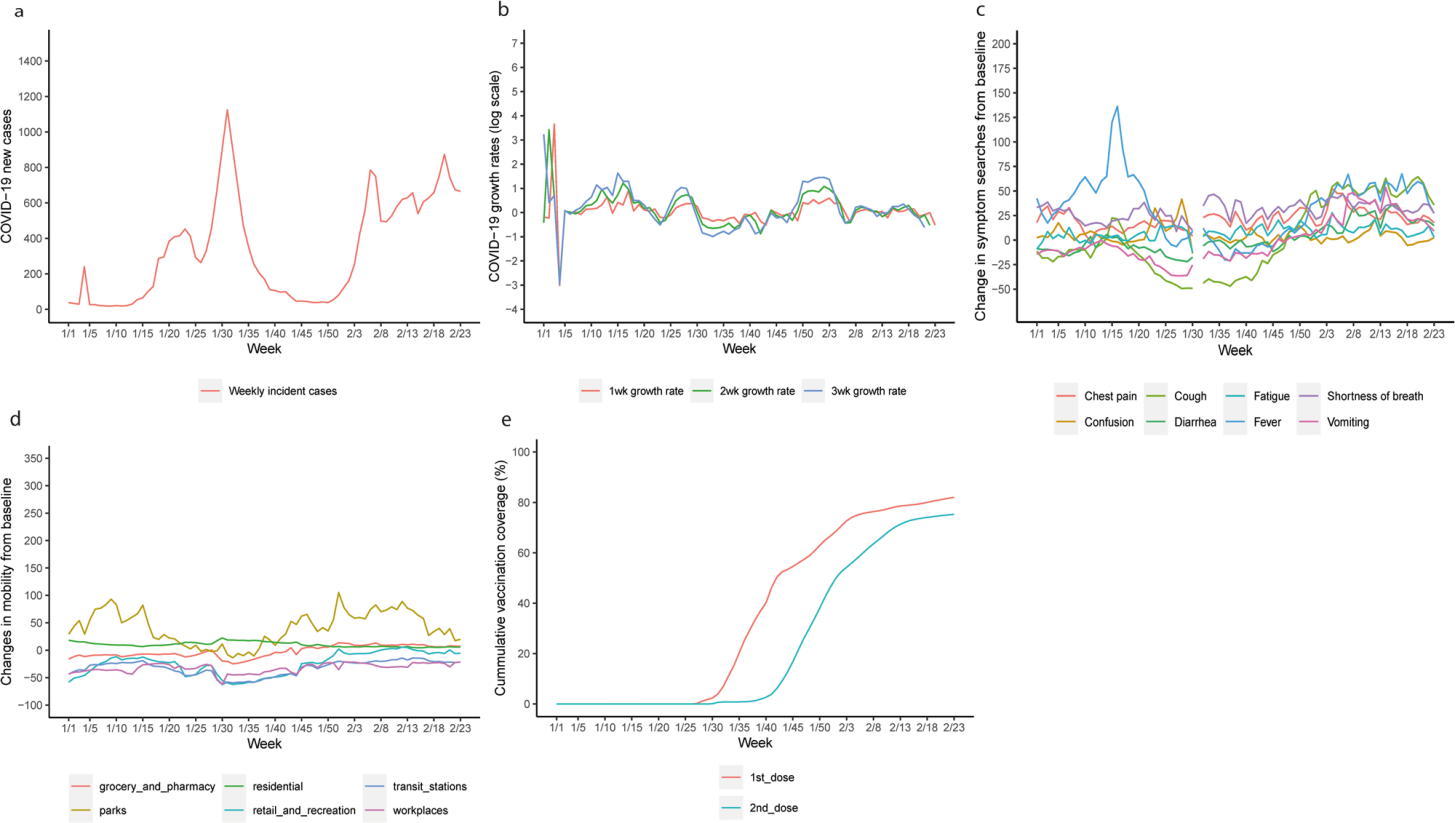
The time series of weekly COVID-19 cases and growth rates, population mobility, symptom searches, and the COVID-19 vaccination coverage. The five panels present various input data included in the analysis aggregated across the UK, including weekly COVID-19 cases (**a**), log scaled weekly COVID-19 growth rates (**b**), Google searches of the eight base symptoms (**c**), Google mobility metrics (**d**) and the COVID-19 vaccination coverage (**e**). Due to the missingness of the baseline searches before the pandemic, we were unable to estimate the changes in Google symptom searches of the eight base symptoms in week 1/31.

We included 367 LTLAs with complete data. The time series of COVID-19 growth rates, mobility, the eight base symptoms and COVID-19 vaccination coverage are in Fig. 2. The COVID-19 growth rates by the collection date of specimen are in Supplementary Fig. 1. COVID-19 growth rates by publication date and by the collection date of specimen showed similar trends over most of the period except in the first five weeks when the curve by publication date changed abruptly, while the curve by the collection date of specimen was flatter (Fig. 2 and Supplementary Fig. 1). All forms of COVID-19 growth rates peaked between weeks 1/10 and 1/20, weeks 1/25 and 1/30, as well as weeks 1/50 and 2/3. The 3-week growth rate showed the largest variation over time, followed by 2-week and 1-week growth rates. The search trends of fever and cough showed a pronounced peak at week 1/15, gradually increased between weeks 1/45 and 2/3, and fluctuated between weeks 2/3 and 2/23; vomiting had a similar trend but varied to a smaller extent. Smaller variations were observed over time for the other five symptoms. Mobility at parks was higher between weeks 1/5 and 1/20, and between 1/45 and 2/18; mobility at retail and recreation areas and transit stations showed a similar trend except for an additional small peak during week 1/25 to 1/30. Time length at residential areas and mobility at grocery and pharmacy was generally stable in the first half of the study period; then time length at residential areas slightly decreased (22.4 in week 1/30 versus 5.7 in week 2/23), while mobility at grocery and pharmacy slightly increased (−19.8 in week 1/30 versus 7.3 in week 2/23). The COVID-19 vaccination coverage increased steadily since the rollout, with the average cumulative uptake of about 82% for the first dose and 75% for the second dose at week 2/23 in population aged 12 and over.

### Optimal model specifications

For the full models, application of time lags and inclusion of additional Google search symptoms (besides the eight base symptoms) improved the predictive accuracy considerably based on the retrospective 4-week MSE (Supplementary Data 1, 2 and Supplementary Figs. 2, 3). Specifically, inclusion of additional Google search symptoms further improved the predictive accuracy after time lags were applied. The optimal lag combination and symptoms varied by checkpoint and timeframe of the prediction. Some symptoms were selected in more than one of the eight checkpoints, including those commonly seen in respiratory infections, i.e., headache, ear pain, otitis, and tonsillitis (Supplementary Data 3–5).

### Prospective prediction performance

**Fig. 3:**
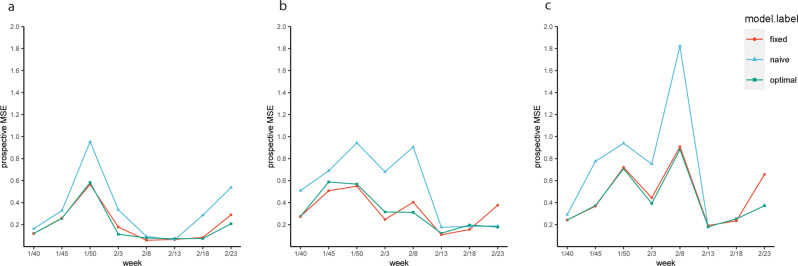
The prospective MSE for predicting COVID-19 growth rates by optimal models and two reference models. The three panels present results for the 1-week (**a**), 2-week (**b**), and 3-week (**c**) prediction of COVID-19 growth rates. MSE: mean squared error. The weeks 1/40, 1/45, 1/50, 2/3, 2/8, 2/13, 2/18, and 2/23 refer to the week of 1st March, 5th April, 10th May, 14th June, 19th July, 30th August, 4th October and 14th November 2021, respectively. The prospective MSE was calculated based on the difference between the observed and predicted COVID-19 growth rate for the week after model selection.

**Fig. 4:**
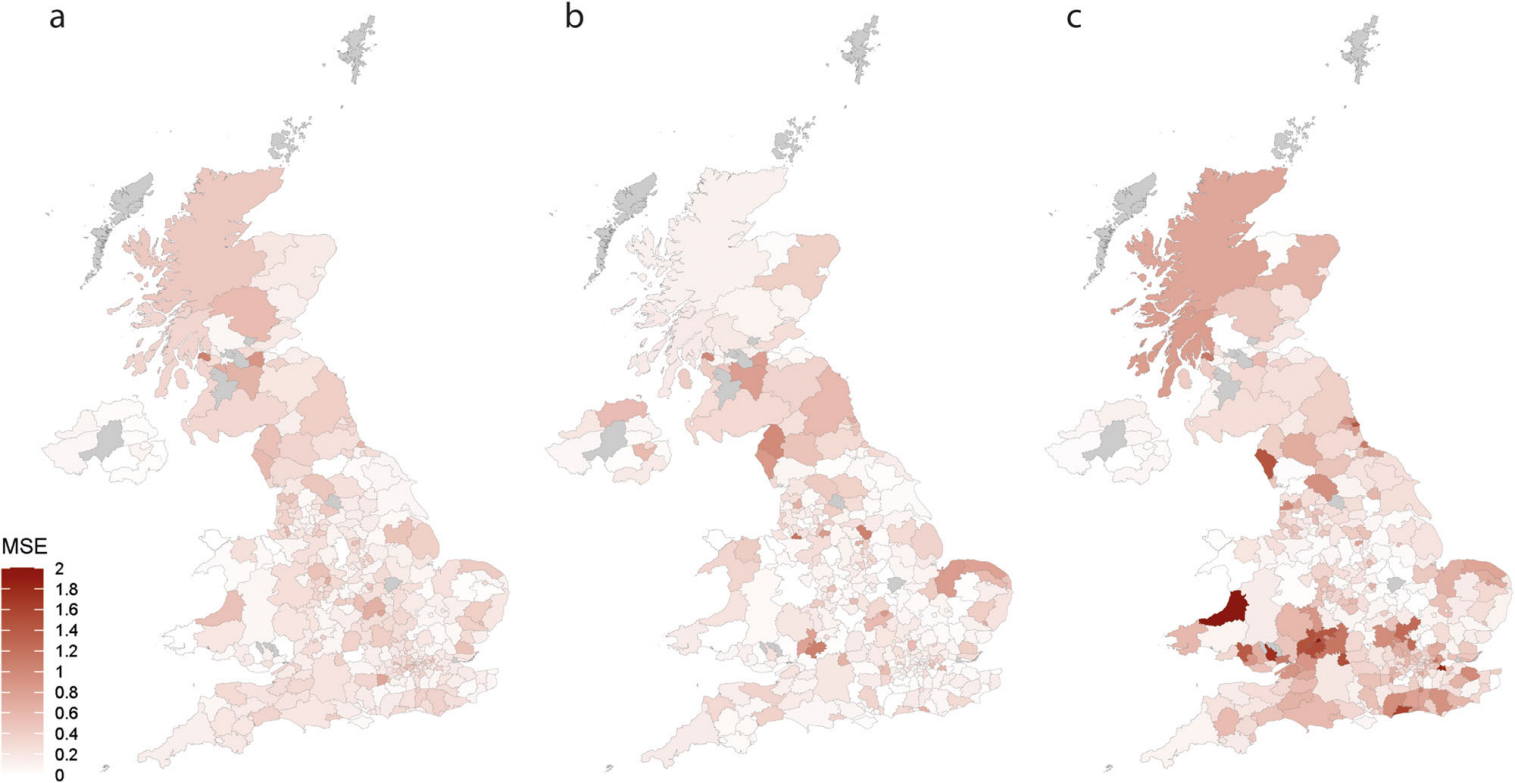
Geographical variations in the prospective MSE by the optimal model for week 2/23. MSE mean squared error. Panel shows the MSE for 1-week growth rate (**a**), 2-week growth rate (**b**), and 3-week growth rate by publication date (**c**).

The prospective predictive accuracy of the optimal full models, measured by prospective MSE, varied over time (i.e., checkpoints), by the timeframe of prediction and between LTLAs. The median MSEs for 1-week, 2-week, and 3-week ahead prediction were 0.12 (IQR: 0.08–0.22), 0.29 (0.19–0.38), and 0.37 (0.25–0.47), respectively, indicating that the prediction accuracy declined with longer prediction timeframe (Fig. 3). Geographical variations were noted in prospective MSE over time. MSEs seemed smaller in the central area of England, while it was larger in Scottish local authorities and south west of England although the patterns were not completely consistent over the eight checkpoints (Fig. 4 and Supplementary Figs. 4–11). Sensitivity analysis that included additional COVID-19-related symptoms did not yield meaningful improvement in prospective predictive accuracy (Supplementary Table 4).

Compared with naïve models, the median reduction in prospective MSE of the optimal models was 21–35% across the three prediction timeframes. For all checkpoints and prediction timeframes, the predictive accuracy of the optimal models was consistently better, with only two exceptions where the two models had similarly low MSEs in week 2/13 for 1-week ahead prediction and in week 2/18 for 2-week ahead prediction. The predictive accuracy did not differ much between the optimal models and fixed-predictors model for most of the checkpoints but at the latest checkpoint (i.e., week 2/23), the optimal model outperformed the fixed-predictors model consistently across the three prediction timeframes (the reduction in MSE ranged 28–58% across the three prediction timeframes) (Fig. 3).

**Fig. 5:**
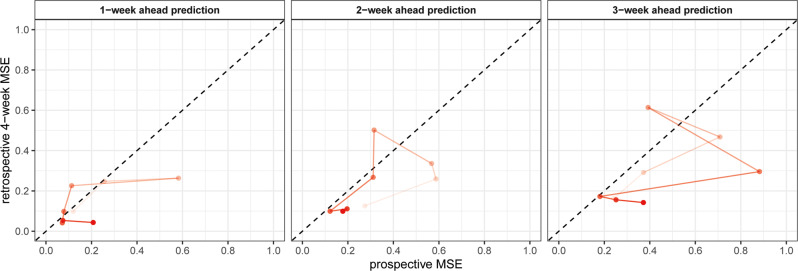
Comparison between retrospective 4-week MSE and prospective MSE for optimal models over different time checkpoints. MSE mean squared error. Darker lines and dots indicate later in time checkpoints. Dashed lines highlight where prospective MSE equals to retrospective 4-week MSE.

Compared with retrospective 4-week MSEs used for model selection, the median inflation of prospective MSE ranged 31–73% across the three prediction timeframes. Despite the inflation, both retrospective 4-week and prospective MSEs followed the same decreasing trend over time and the absolute difference between the two MSEs diminished after the first 3–4 checkpoints (Fig. 5).

## Discussion

Our results show that our dynamic supervised machine-learning algorithm, allowing the optimal model specification to vary based on the latest data, can help improve short-term predictions of the weekly growth rates in the COVID-19 cases in the UK. The optimal predictions were informed by different model specifications (e.g., different symptom search queries) over time. Compared with the naïve model, the predictions, optimised on the digital metrics, were more accurate on most occasions and managed to reduce MSE by 21–35%. During the times when the COVID-19 cases increased rapidly (around week 2/3), the models with predictors maintained increased predictive accuracy compared with the naïve models. In addition, we present a publicly accessible web application COVIDPredLTLA developed to update the predictions of COVID-19 growth rates by LTLA using near real-time data.

We focused on predicting the growth rate of COVID-19 cases in our models. We consider that the COVID-19 growth rate could better inform the implementation and relaxation of the control strategies compared with predicted COVID-19 cases^4^. Fitting, prediction and assessment are addressed at LTLA resolution; LTLA-specific predictions could be used to inform control strategies at fine geographical level. We highlight the consistently better performance of the optimal models compared with naïve models, including the time period (around week 2/3) when there were substantial rise in the COVID-19 growth rates, likely due to the rapid circulation of the delta variant in the UK^29–31^. The model performance suggests that our model shows promise in predicting rapid changes in the COVID-19 cases. The model could be helpful when there are epidemic growths in future that are associated with the emergence and circulation of new variants and lineages, e.g., the omicron variant with high potential for evading or eroding existing infection- and vaccine-derived immunity, which became the predominant circulating variant at the time of writing^32^. As we applied a flexible algorithm for selection of symptoms, our model has the potential for adapting itself to the changes in the spectrum of symptoms over time^17^, e.g., due to the circulation of new variants and the mass rollout of the vaccination programme. Compared with the fixed-predictors model, our dynamic model is more flexible in nature and showed advantages in predictive accuracy after a few months of updating from the original model.

As expected, owing to the absence of input digital metrics in the most recent timeframe, the predictive accuracy dropped for longer prediction timeframe for all models in our analysis, which is similar to a recent COVID-19 forecasting study in Germany and Poland that utilised different a modelling approach^33^. This highlights the challenge in forecasting respiratory infectious disease epidemics such as COVID-19 that are highly driven by most recent population behaviours.

We acknowledge several potential limitations. The accuracy of real-time nowcasts and forecasts are challenged by delayed data^1^. We choose the COVID-19 cases by publication date rather than the date of collection of specimen as the main outcome. When using date of collection of specimen as the input data for analysis, historical data may have the strength of accounting for testing delays, but the real-time data are usually incomplete with some cases awaiting to be added in the next few days. This type of reporting delays causes an artificial decrease in the real-time COVID-19 cases. Google mobility and symptom searches are further delayed for several days (4–5 days) relative to the COVID-19 cases. Secondly, the real trend of the cases could have been blurred by changes in the testing practice and this impact is difficult to quantify. In the UK, the testing programmes were scaled up in the first few weeks of the study period (June–July 2020)^34^. Since then the daily cases include results from a wider population in addition to those with clinical needs and healthcare workers. In England, the cases include positive polymerase chain reaction (PCR) test results, positive rapid lateral flow test results confirmed with PCR tests taken within 72 h, and positive loop-mediated isothermal amplification test results; the other nations only include positive PCR results^34^. Thirdly, certain age subgroups (e.g., older adults) may be underrepresented in the Google mobility data and symptom search trends due to their limited access to the internet. Additionally, we noticed a spike in Google searches for fever in the first quarter of the study period. The spike could be a signal of subsequent increase in cases. Since then the subsequent peaks in fever were notably lower; it could be because public were less concerned in fever or less had fever after vaccination^15,17^. We did not include dysgeusia and anosmia in the main model due to incompleteness of Google search data on the two symptoms; among the LTLAs with complete data on dysgeusia and anosmia, the MSEs were similar when including them (Supplementary Table 4). Although the prediction uncertainty intervals are provided in the online web application, the uncertainty might only reflect the variations in model parameters (e.g., not reflecting the variability of input data, which is difficult to quantify) and should be interpreted with caution. We have not accounted for other prevention measures, such as wearing facemasks and hand hygiene, and changes in the climate factors^35^, which might also have influenced the COVID-19 epidemic. Due to data scarcity on the proportion of different SARS-CoV-2 variants by LTLA, we were unable to assess the model predictability specifically by variant.

## Conclusion

We developed and validated a dynamic supervised machine-learning modelling framework that predicted local weekly growth rates in the COVID-19 cases in the UK by integrating digital metrics of Google mobility and search trends, and the COVID-19 vaccination coverage. Our models were validated against reference models in the time period when there were rapid increases associated with the emergence and circulation of the delta variant, and thus showed promises in predicting future rapid changes in the COVID-19 cases. We will continue monitoring the model performance and updating all the predictions regularly using new data on a publically accessible web application, which could assist decision-making at local authorities during the ongoing pandemic.

## Supplementary information

Supplmentary Information

Description of Additional Supplementary Files

Supplmentary Data 1

Supplmentary Data 2

Supplmentary Data 3

Supplmentary Data 4

Supplmentary Data 5

Supplmentary Data 6

Supplmentary Data 7

Supplmentary Data 8

Supplmentary Data 9

Reporting Summary

## Data Availability

All the data used in this study are publicly available: the Google Search Trends symptoms dataset https://pair-code.github.io/covid19_symptom_dataset/?country=UK; the Google Community Mobility Reports https://www.google.com/covid19/mobility/; COVID-19 vaccination coverage and COVID-19 cases for the UK https://coronavirus.data.gov.uk/details/download. The source data for Figs. 2–5 are available as Supplementary Data 6–9.

## Code availability

The source R codes for constructing the model are available at Zenodo^36^.
