# Supplementary material for: Short-term local predictions of COVID-19 in the United Kingdom using dynamic supervised machine learning algorithms
Source: Commun Med (Lond). 2022 Sep 24;2:119. doi: 10.1038/s43856-022-00184-7 (PMC9509378; doi:10.1038/s43856-022-00184-7)
Supplement: Supplementary file 1 — Supplmentary Information [file 43856_2022_184_MOESM1_ESM.pdf]

Supplementary information for "Short-term local predictions of COVID-19 in the United Kingdom using dynamic supervised machine learning algorithms"

Xin Wang, Yijia Dong, William David Thompson, Harish Nair, You Li

Contents

Supplementary methods ... 3
Step 1. Searching for the optimal lags in predictors using the base model ... 3
Step 2. Searching for the optimal list of Google symptoms predictors ... 3
Step 3. Additional sensitivity analyses by removing one of the three datasets ... 4
Na ïve models ... 5
Estimating the retrospective 4-week MSE for model selection ... 5
Estimating the prospective 1-week MSE for prediction performance ... 5
Summary of COVID-19 growth rates by the collection date of specimen ... 6
Model selection ... 7
Lag combinations by prediction timeframe ... 7
Overview of model comparison results ... 8
Prospective prediction performance ... 9
Sensitivity analysis - revised list of symptoms ... 9
Prospective MSEs by LTLAs using the optimal models ... 10

Supplementary methods

We conducted a complete modelling development and validation process for each of the six outcomes as outlined in the main text. Below we use one-week growth rate prediction as an example to illustrate all the steps of the process.

The process included fitting and predicting that are done at LTLA resolution. We used the log-linear regression to fit the data, and considered three sets of candidate predictors: vaccination (two variables), Google mobility (six variables), and Google symptoms (eight base variables + additional variables).

Step 1. Searching for the optimal lags in predictors using the base model

As described in Methods, our starting point was the base model that included LTLA, COVID-19 vaccination (two variables), Google mobility (six variables for six locations) and the eight base variables from Google symptoms. Then we searched for the optimal time lags for each of the datasets by comparing the prediction errors (details are provided below) of the following candidate models:

Supplementary Table 1 *List of candidate models*

**Model (A total of 64 models)**	**Outcome**	**Predictors**		
		**Vaccination: XV1 and XV2**	**Google Mobility: XM1, XM2, …, XM6**	**Google Symptom: XS1, XS2, …, XS10**
Lag: XV=0, XM=0, XS=0	$Y1p_t$	$XV1_t$, $XV2_t$	$XM1_t$, $XM2_t$, …, $XM6_t$	$XS1_t$, $XS2_t$, …, $XS10_t$
Lag: XV=1, XM=0, XS=0	$Y1p_t$	$XV1_{t-1}$, $XV2_{t-1}$	$XM1_t$, $XM2_t$, …, $XM6_t$	$XS1_t$, $XS2_t$, …, $XS10_t$
Lag: XV=2, XM=0, XS=0	$Y1p_t$	$XV1_{t-2}$, $XV2_{t-2}$	$XM1_t$, $XM2_t$, …, $XM6_t$	$XS1_t$, $XS2_t$, …, $XS10_t$
Lag: XV=3, XM=0, XS=0	$Y1p_t$	$XV1_{t-3}$, $XV2_{t-3}$	$XM1_t$, $XM2_t$, …, $XM6_t$	$XS1_t$, $XS2_t$, …, $XS10_t$
Lag: XV=0, XM=1, XS=0	$Y1p_t$	$XV1_t$, $XV2_t$	$XM1_{t-1}$, $XM2_{t-1}$, …, $XM6_{t-1}$	$XS1_t$, $XS2_t$, …, $XS10_t$
Lag: XV=1, XM=1, XS=0	$Y1p_t$	$XV1_{t-1}$, $XV2_{t-1}$	$XM1_{t-1}$, $XM2_{t-1}$, …, $XM6_{t-1}$	$XS1_t$, $XS2_t$, …, $XS10_t$
Lag: XV=2, XM=1, XS=0	$Y1p_t$	$XV1_{t-2}$, $XV2_{t-2}$	$XM1_{t-1}$, $XM2_{t-1}$, …, $XM6_{t-1}$	$XS1_t$, $XS2_t$, …, $XS10_t$
Lag: XV=3, XM=1, XS=0	$Y1p_t$	$XV1_{t-3}$, $XV2_{t-3}$	$XM1_{t-1}$, $XM2_{t-1}$, …, $XM6_{t-1}$	$XS1_t$, $XS2_t$, …, $XS10_t$
Lag: XV=0, XM=2, XS=0	$Y1p_t$	$XV1_t$, $XV2_t$	$XM1_{t-2}$, $XM2_{t-2}$, …, $XM6_{t-2}$	$XS1_t$, $XS2_t$, …, $XS10_t$
Lag: XV=1, XM=2, XS=0	$Y1p_t$	$XV1_{t-1}$, $XV2_{t-1}$	$XM1_{t-2}$, $XM2_{t-2}$, …, $XM6_{t-2}$	$XS1_t$, $XS2_t$, …, $XS10_t$
Lag: XV=2, XM=2, XS=0	$Y1p_t$	$XV1_{t-2}$, $XV2_{t-2}$	$XM1_{t-2}$, $XM2_{t-2}$, …, $XM6_{t-2}$	$XS1_t$, $XS2_t$, …, $XS10_t$
Lag: XV=3, XM=2, XS=0	$Y1p_t$	$XV1_{t-3}$, $XV2_{t-3}$	$XM1_{t-2}$, $XM2_{t-2}$, …, $XM6_{t-2}$	$XS1_t$, $XS2_t$, …, $XS10_t$
Lag: XV=0, XM=0, XS=1	$Y1p_t$	$XV1_t$, $XV2_t$	$XM1_t$, $XM2_t$, …, $XM6_t$	$XS1_{t-1}$, $XS2_{t-1}$, …, $XS10_{t-1}$
Lag: XV=1, XM=0, XS=1	$Y1p_t$	$XV1_{t-1}$, $XV2_{t-1}$	$XM1_t$, $XM2_t$, …, $XM6_t$	$XS1_{t-1}$, $XS2_{t-1}$, …, $XS10_{t-1}$
Lag: XV=2, XM=0, XS=1	$Y1p_t$	$XV1_{t-2}$, $XV2_{t-2}$	$XM1_t$, $XM2_t$, …, $XM6_t$	$XS1_{t-1}$, $XS2_{t-1}$, …, $XS10_{t-1}$
Lag: XV=3, XM=0, XS=1	$Y1p_t$	$XV1_{t-3}$, $XV2_{t-3}$	$XM1_t$, $XM2_t$, …, $XM6_t$	$XS1_{t-1}$, $XS2_{t-1}$, …, $XS10_{t-1}$
…	…	…	…	…
Lag: XV=0, XM=3, XS=3	$Y1p_t$	$XV1_t$, $XV2_t$	$XM1_{t-3}$, $XM2_{t-3}$, …, $XM6_{t-3}$	$XS1_{t-3}$, $XS2_{t-3}$, …, $XS10_{t-3}$
Lag: XV=1, XM=3, XS=3	$Y1p_t$	$XV1_{t-1}$, $XV2_{t-1}$	$XM1_{t-3}$, $XM2_{t-3}$, …, $XM6_{t-3}$	$XS1_{t-3}$, $XS2_{t-3}$, …, $XS10_{t-3}$
Lag: XV=2, XM=3, XS=3	$Y1p_t$	$XV1_{t-2}$, $XV2_{t-2}$	$XM1_{t-3}$, $XM2_{t-3}$, …, $XM6_{t-3}$	$XS1_{t-3}$, $XS2_{t-3}$, …, $XS10_{t-3}$
Lag: XV=3, XM=3, XS=3	$Y1p_t$	$XV1_{t-3}$, $XV2_{t-3}$	$XM1_{t-3}$, $XM2_{t-3}$, …, $XM6_{t-3}$	$XS1_{t-3}$, $XS2_{t-3}$, …, $XS10_{t-3}$

XV, XM and XS refer to the time lag (in weeks) for vaccination, mobility and symptom search data.

Step 2. Searching for the optimal list of Google symptoms predictors

Next we kept the time lags as the optimal lags obtained from Step 1, and tested whether adding additional symptoms from the rest of the Google symptoms (165 symptoms) improved the model by assessing prediction errors (details are provided below).

As described in the Methods, there were eight variables for the search trends of common symptoms for COVID-19 (cough, fever, fatigue, diarrhoea, vomiting, shortness of breath, confusion, and chest pain) in the base model. Then we added one additional symptom from the rest symptoms each time (i.e. the model included 8 base symptoms plus 1 additional symptom) and compared the prediction errors. If none of the models outperformed the original model (here being the base model), then that concluded Step 2. Otherwise, we selected the new best performing model as the new "base model" and repeated this process until completing the assessment for all the symptoms.

Step 3. Additional sensitivity analyses by removing one of the three datasets

We kept the optimal lags and symptoms obtained from Step 1 and 2, and tested whether removing any one or two of the three sets of predictors improved the model by assessing prediction errors (details are provided below).

Naïve models

Supplementary Table 2 Lagged naïve models

Lags	Predicts for week t
0 week	$Ypredicted_t = Yobserved_{t-1}$
1 week	$Ypredicted_t = Yobserved_{t-2}$
2 weeks	$Ypredicted_t = Yobserved_{t-3}$
3 weeks	$Ypredicted_t = Yobserved_{t-4}$

Estimating the retrospective 4-week MSE for model selection

As described above, we assessed a model based on retrospective prediction errors. We used the retrospective 4-week mean squared errors (MSEs) of prediction for assessment. For the ease of presentation, we defined week 1 as the starting week of our data (i.e. w/c 1-June-2020) and defined week t as the "current" week (the most recent week with complete COVID-19 cases). When predicting the changes in the COVID-19 cases in week t+1, we assumed that the COVID-19 cases were only available until week t (similar to the scenario of real-time prediction). Thus, the 4-week period is related to the length of the prediction timeframe. The table below shows the estimation of the 4-week MSE.

Each row below in Table S2 corresponds a model using the "historical data" (i.e. in green) to predict the Y in the week in red. For each model, we calculated the average difference (e.g. D1 for model 1) between $Ypredicted$ (log scaled) and $Yobserved$ (log scaled) across LTLA. The square value of D1 (i.e. $D1^2$) is the MSE for this model. We calculated the 4-week MSE by averaging $D1^2$, $D2^2$, $D3^2$ and $D4^2$. The model that had the minimum 4-week MSE was the optimal model, and was used to predict for the target week.

Estimating the prospective 1-week MSE for prediction performance

Next, we assessed the prediction performance of the different selected models, by predicting the COVID-19 growth rate for the next target week.

Supplementary Table 3 *Estimating the retrospective 4-week average MSE and prospective one-week MSE when predicting for week t, using the prediction timeframe of i.*

Model	1	2	…	21	22	…	t-*i*-3	t-*i*-2	t-*i*-1	t-*i*	*t-i+1*
5											
4											
3											
2											
1											

Green: Training dataset. Yellow: "retrospective" validation (for model selection). Red: "prospective" validation (for assessing prediction performance)

Summary of COVID-19 growth rates by the collection date of specimen

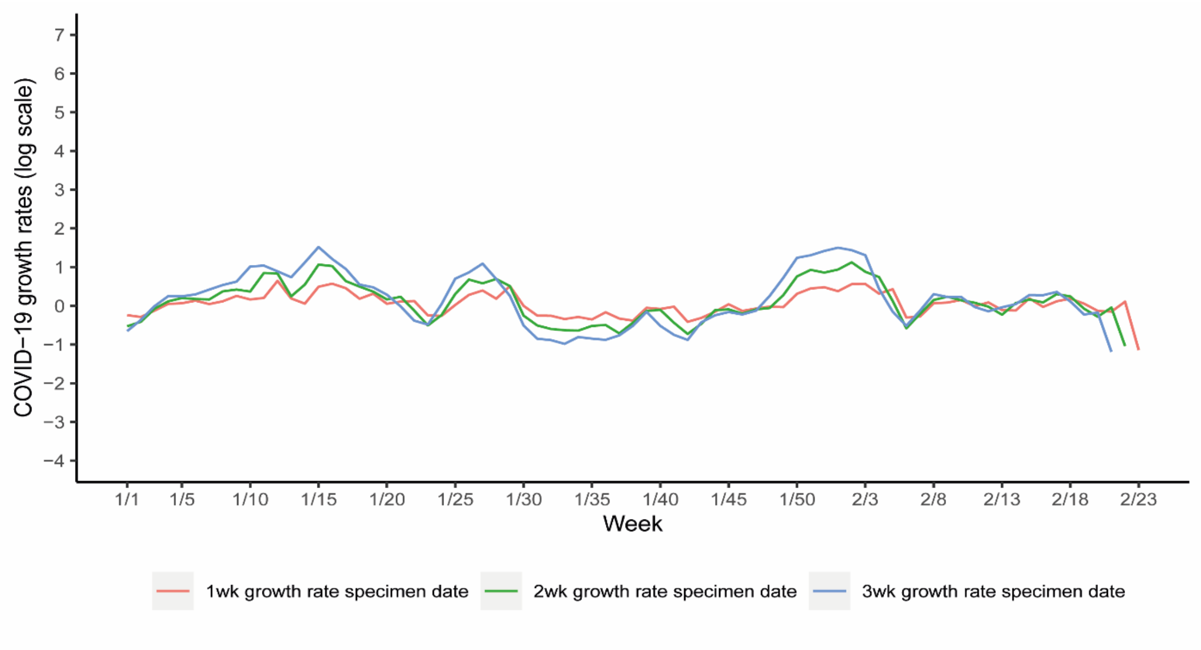

Supplementary Figure 1 *The time series of COVID-19 growth rates by the collection date of specimen.*

Model selection

Lag combinations by prediction timeframe

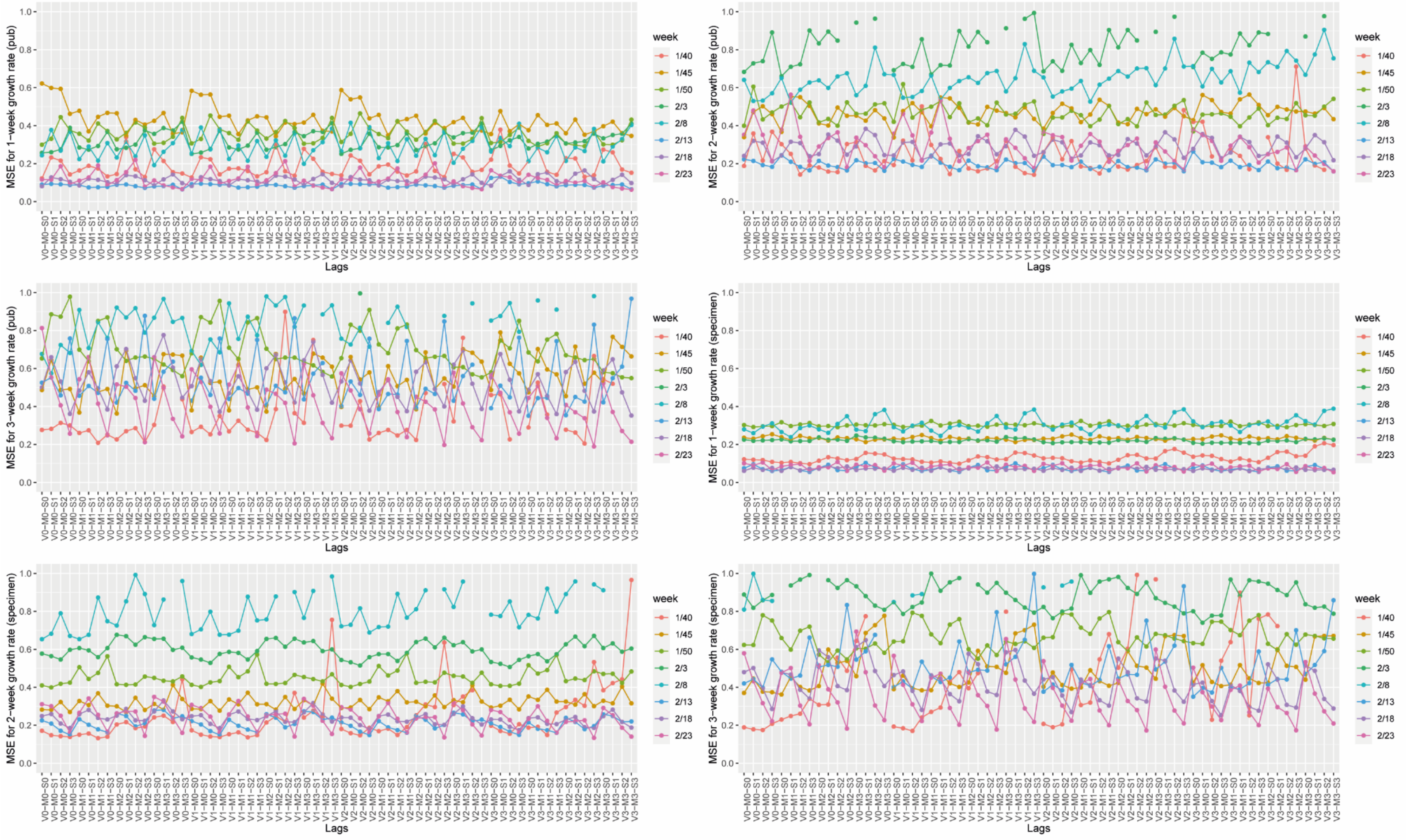

Supplementary Figure 2 The retrospective 4-week MSEs for different time lag combinations by week and prediction timeframe.

MSE: mean squared error. Models include LTLA, mobility, search trends of the eight symptoms and the vaccination coverage. V0, V1, V2 and V3: 0-3 weeks' lags in vaccination coverage. M0, M1, M2 and M3: 0-3 weeks in mobility. S0, S1, S2 and S3: 0-3 weeks' lags in symptom search trends. Pub: by publication date. Specimen: by the date of collection of specimen.

Overview of model comparison results

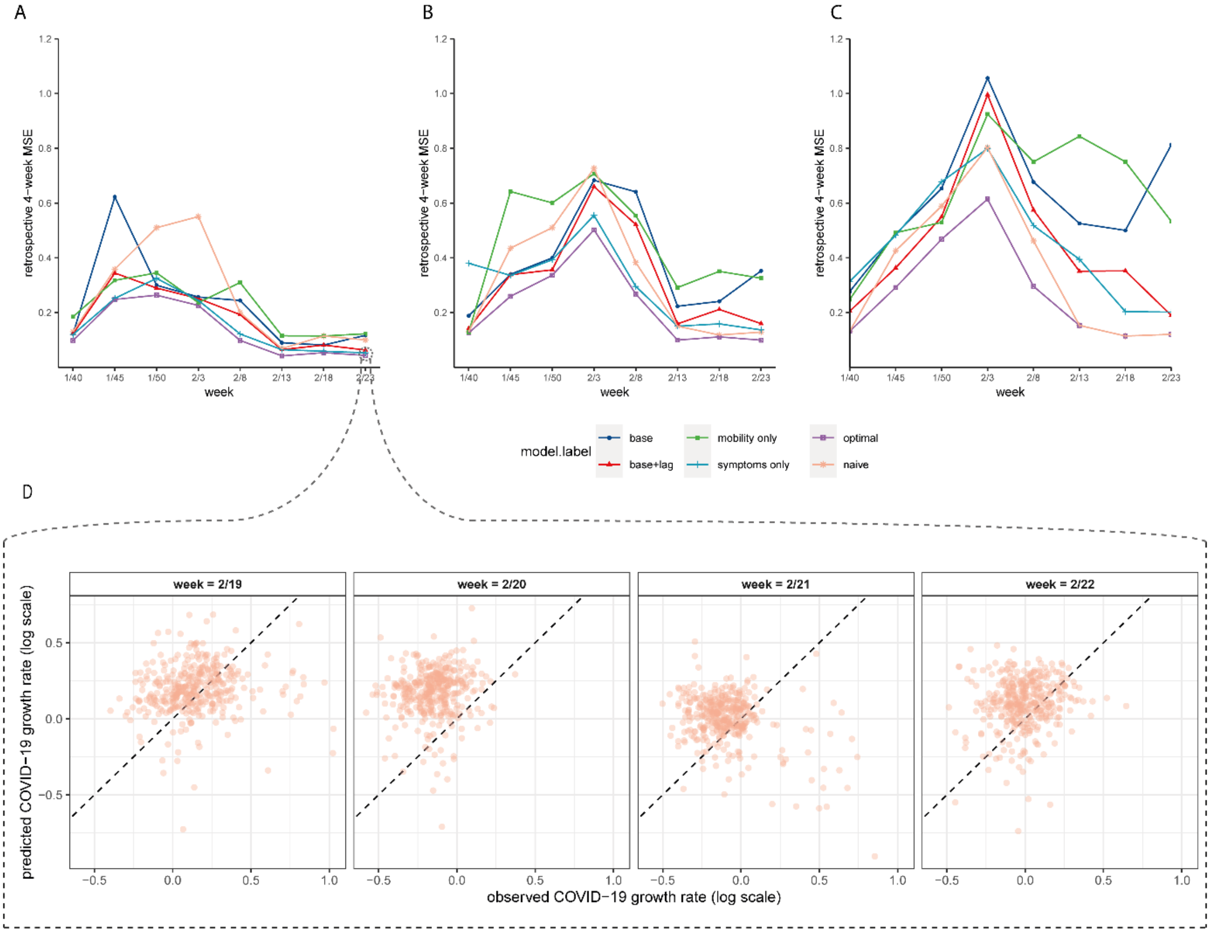

Supplementary Figure 3. *The retrospective 4-week MSE for the prediction of COVID-19 growth rates by na ïve models and full models, and comparison between observation and prediction for the week 2/23.*

Panel (A), (B) and (C) present the retrospective 4-week MSE for the 1-week, 2-week, and 3-week prediction of COVID-19 growth rates by na ïve models and full models. Panel (D) presents the comparison between observation and prediction for the week 2/23. MSE: mean squared error. The weeks 1/40, 1/45, 1/50, 2/3, 2/8, 2/13, 2/18 and 2/23 refer to the week of 1st March, 5th April, 10th May, 14th June, 19th July, 30th August, 4th October and 14th November 2021, respectively. The retrospective 4-week MSE refers to the average MSE over the previous four weeks for the same candidate model. In Panel D, a break-down of MSE for each week is presented for the week 2/23; individual dots denote individual lower-tier local authorities.

Prospective prediction performance

Sensitivity analysis - revised list of symptoms

In the sensitivity analysis, “dysgeusia”, “anosmia”, “headache”, “nasal congestion” and “sore throat” were added to the base model to test whether including these symptoms improved the predictive accuracy.

The prospective 1-week MSE of the sensitivity analyses and those of the main analysis for week 2/23 are shown below.

Supplementary Table 4 *Comparing the sensitivity analyses with the main analysis, week 2/23 by publication date.*[*]

	1-week growth rate (prospective MSE)	2-week growth rate (prospective MSE)	3-week growth rate (prospective MSE)
Week 2/23			
Sensitivity analysis 1: add base symptoms	0.22	0.20	0.28
Main analysis: base symptoms in the same LTLAs	0.22	0.14	0.33

[*] MSE: mean squared error.

Prospective MSEs by LTLAs using the optimal models

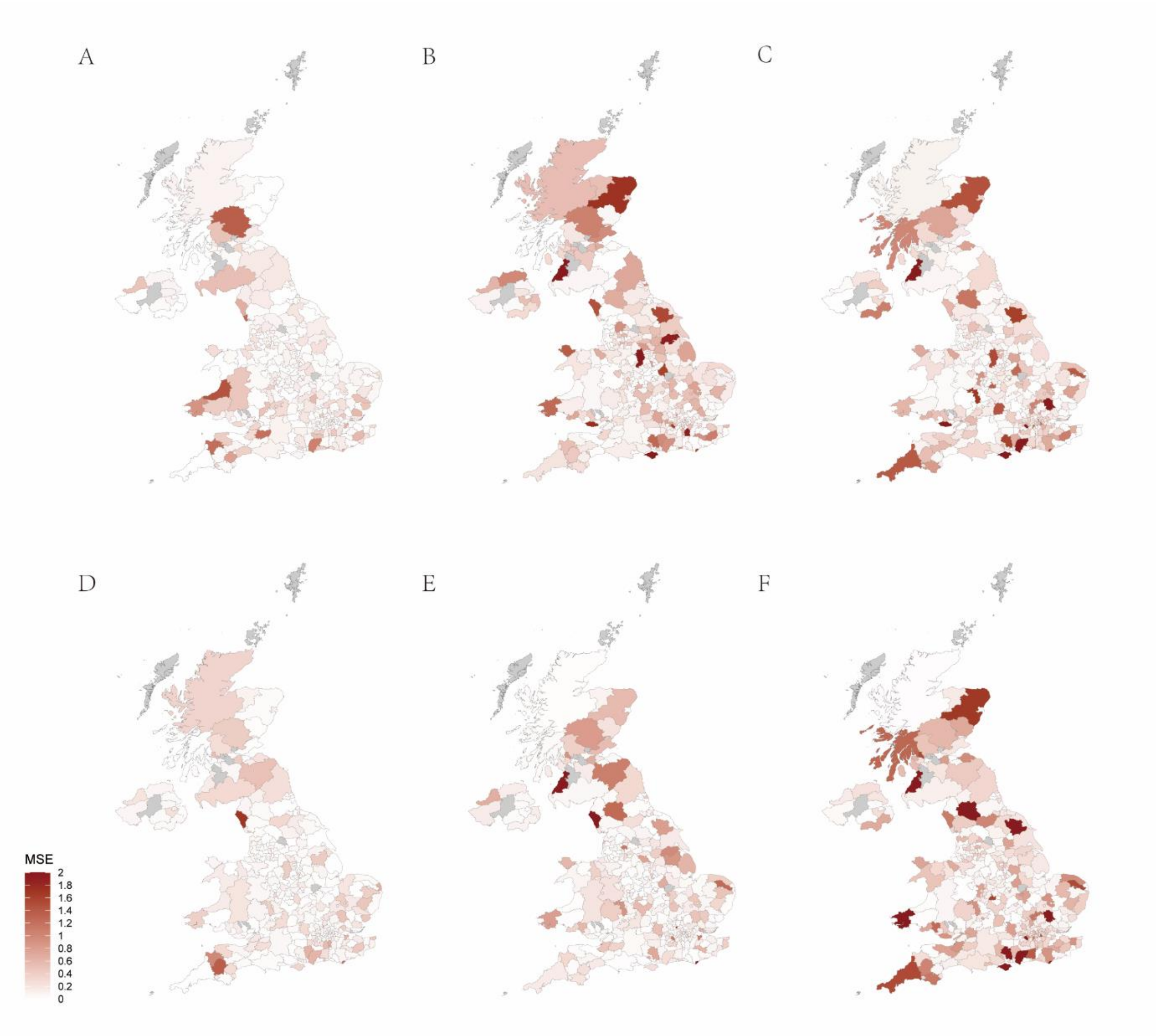

Supplementary Figure 4 Prospective 1-week MSEs by prediction timeframe, week 1/40.

MSE: mean squared error. Panels: 1-week growth rate (A), 2-week growth rate (B) and 3-week growth rate (C) by publication date; 1-week growth rate (D), 2-week growth rate (E) and 3-week growth rate (F) by the collection date of specimen.

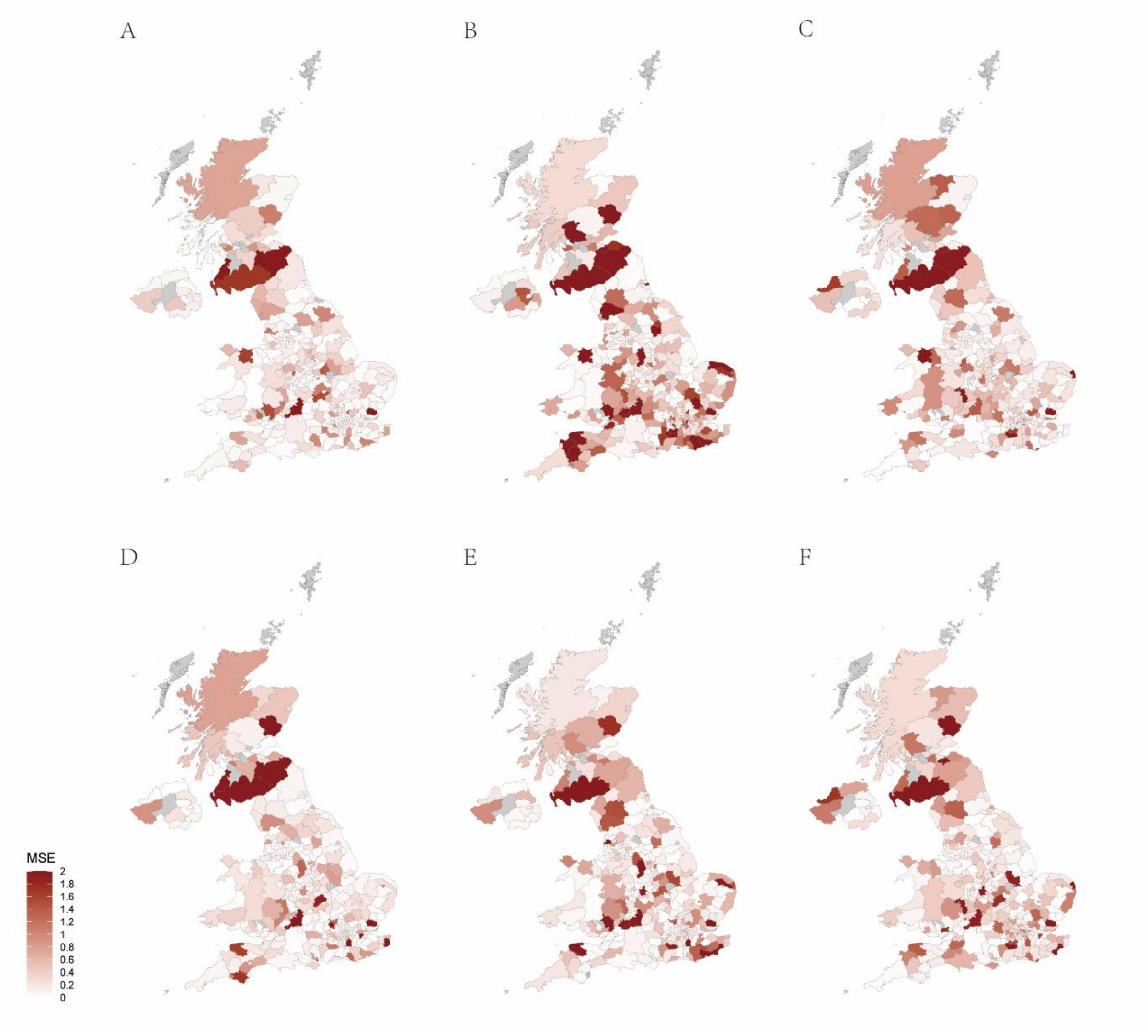

Supplementary Figure 5 Prospective 1-week MSEs by prediction timeframe, week 1/45.

MSE: mean squared error. Panels: 1-week growth rate (A), 2-week growth rate (B) and 3-week growth rate (C) by publication date; 1-week growth rate (D), 2-week growth rate (E) and 3-week growth rate (F) by the collection date of specimen.

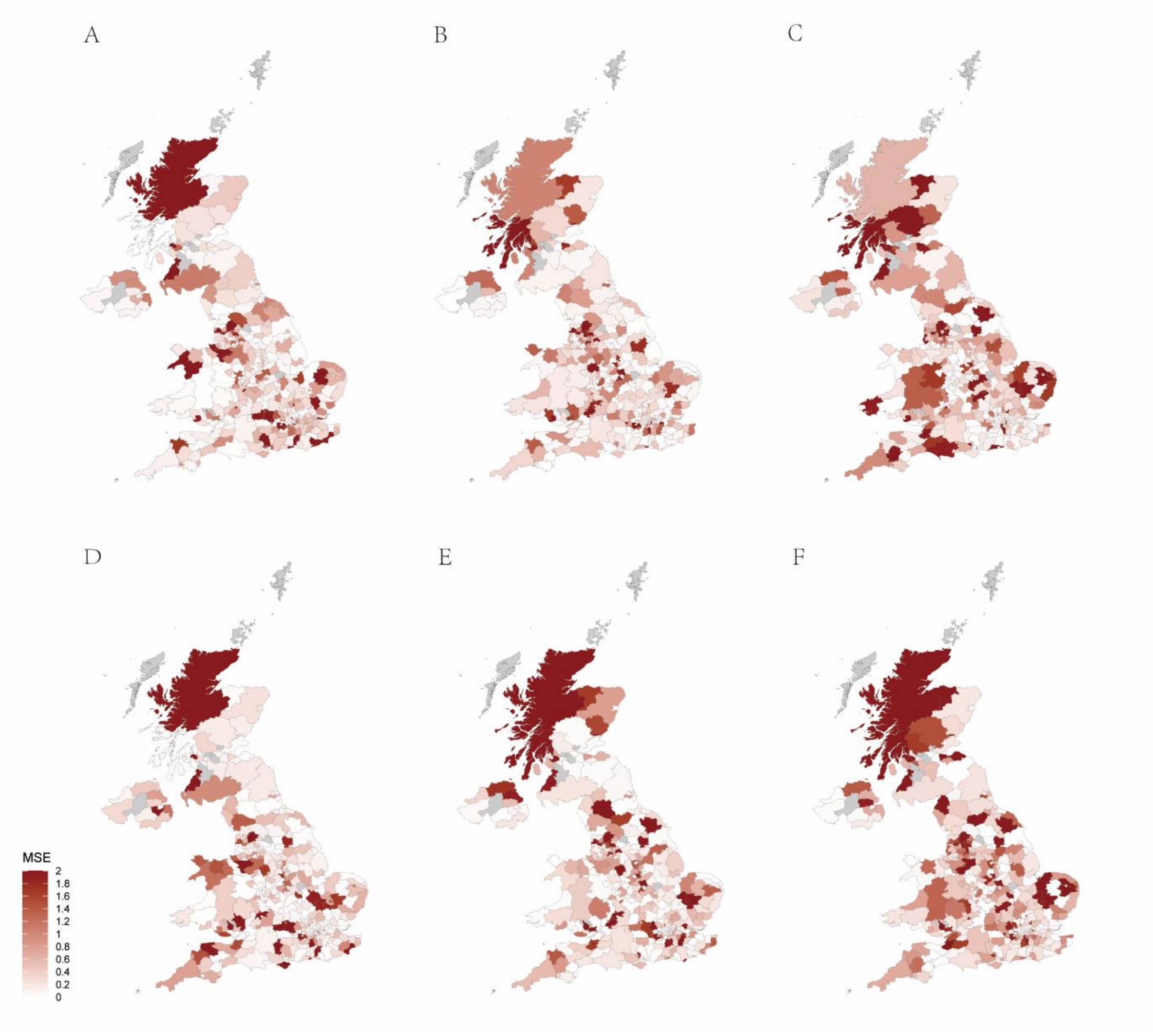

Supplementary Figure 6 Prospective 1-week MSEs by prediction timeframe, week 1/50.

MSE: mean squared error. Panels: 1-week growth rate (A), 2-week growth rate (B) and 3-week growth rate (C) by publication date; 1-week growth rate (D), 2-week growth rate (E) and 3-week growth rate (F) by the collection date of specimen.

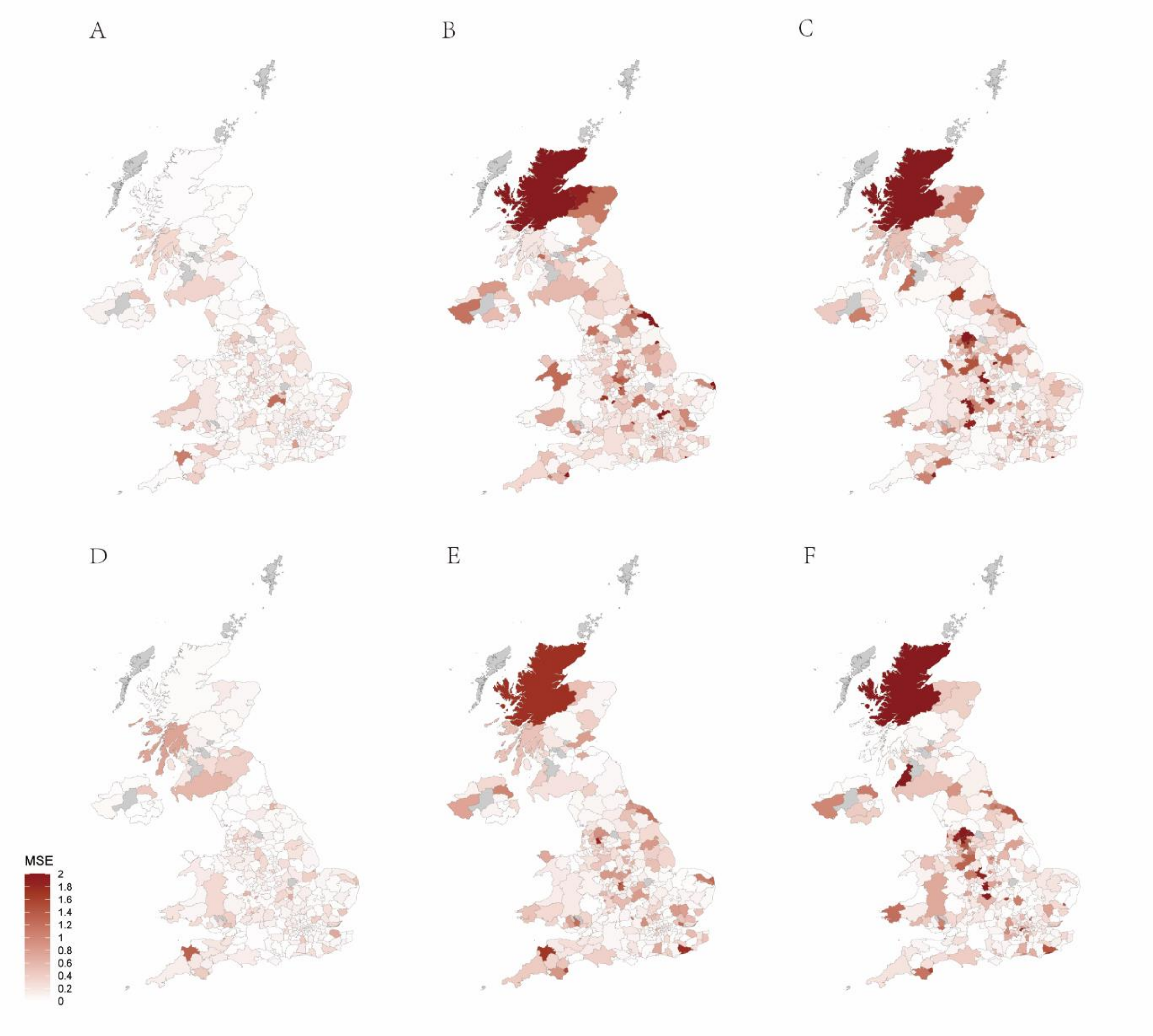

Supplementary Figure 7 Prospective 1-week MSEs by prediction timeframe, week 2/3.

MSE: mean squared error. Panels: 1-week growth rate (A), 2-week growth rate (B) and 3-week growth rate (C) by publication date; 1-week growth rate (D), 2-week growth rate (E) and 3-week growth rate (F) by the collection date of specimen.

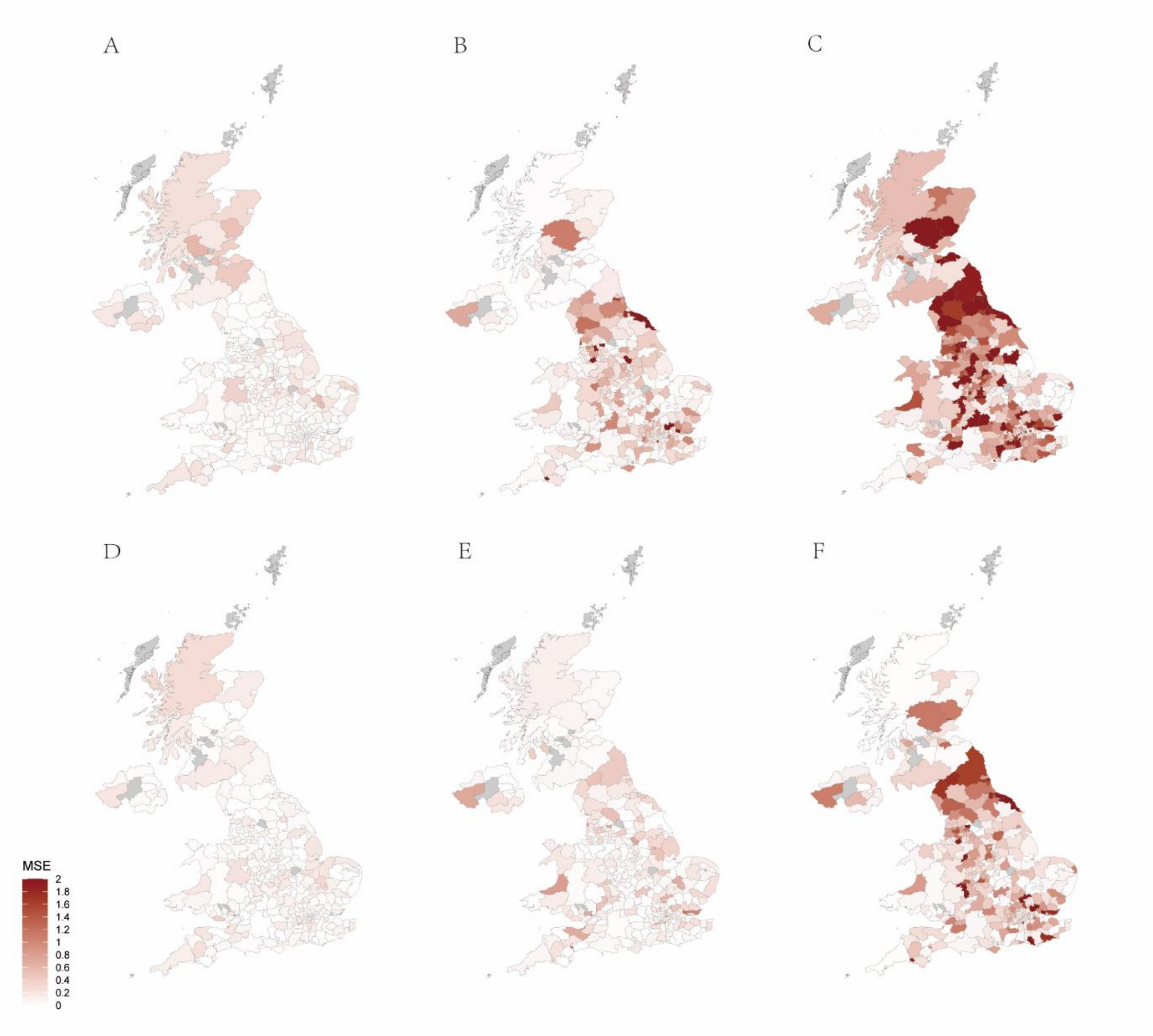

Supplementary Figure 8 Prospective 1-week MSEs by prediction timeframe, week 2/8.

MSE: mean squared error. Panels: 1-week growth rate (A), 2-week growth rate (B) and 3-week growth rate (C) by publication date; 1-week growth rate (D), 2-week growth rate (E) and 3-week growth rate (F) by the collection date of specimen.

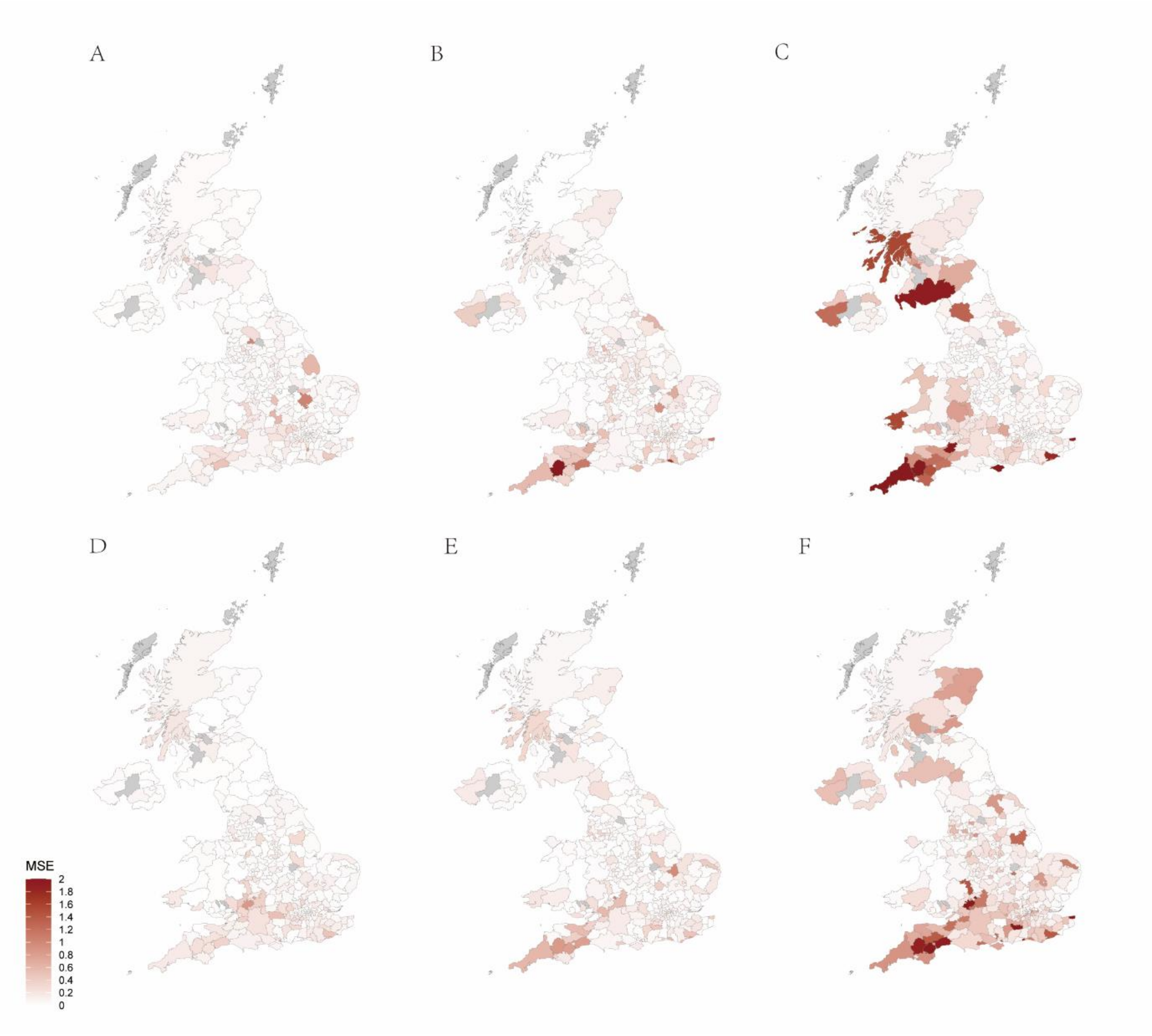

Supplementary Figure 9 Prospective 1-week MSEs by prediction timeframe, week 2/13.

MSE: mean squared error. Panels: 1-week growth rate (A), 2-week growth rate (B) and 3-week growth rate (C) by publication date; 1-week growth rate (D), 2-week growth rate (E) and 3-week growth rate (F) by the collection date of specimen.

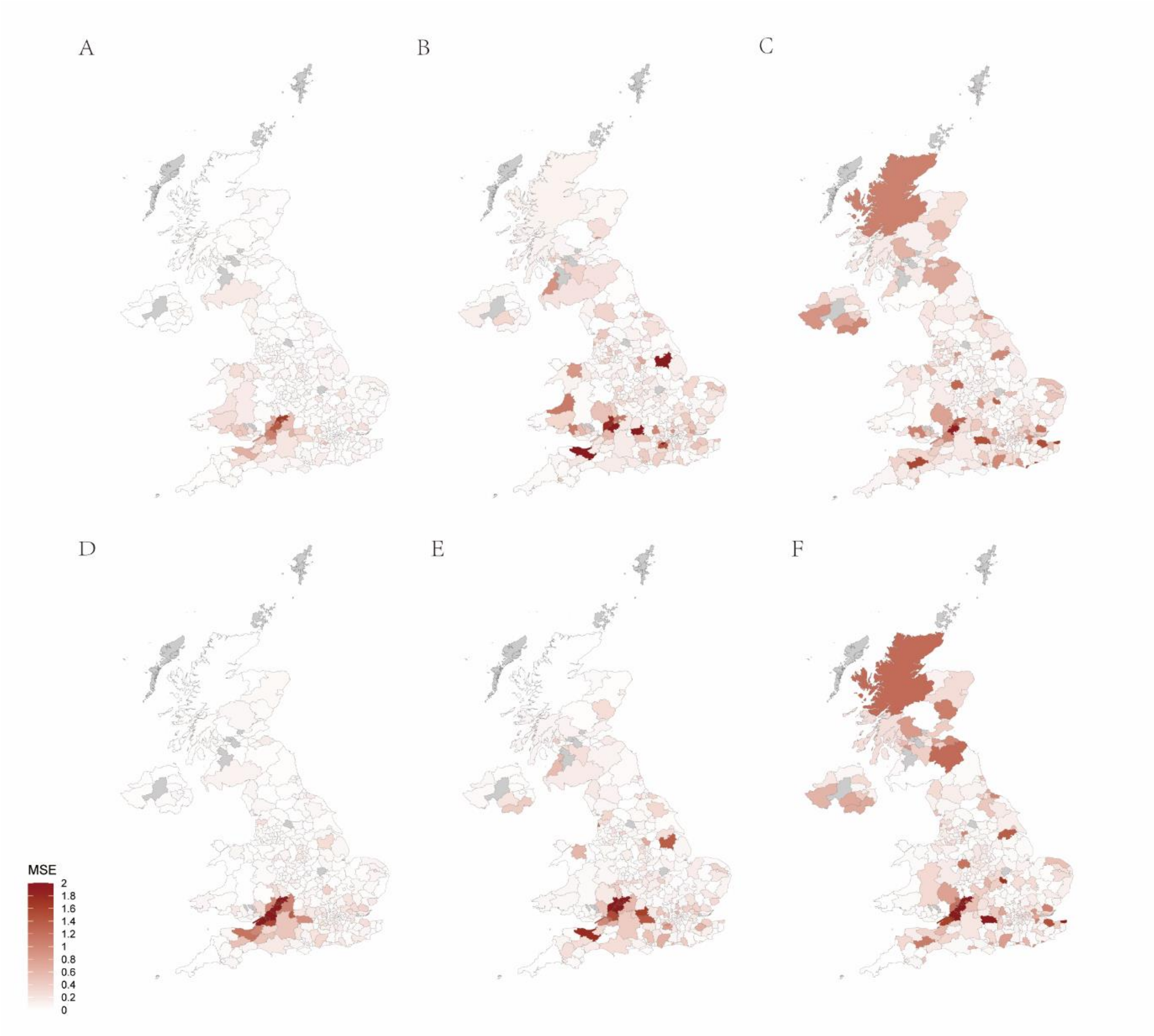

Supplementary Figure 10 Prospective 1-week MSEs by prediction timeframe, week 2/18.

MSE: mean squared error. Panels: 1-week growth rate (A), 2-week growth rate (B) and 3-week growth rate (C) by publication date; 1-week growth rate (D), 2-week growth rate (E) and 3-week growth rate (F) by the collection date of specimen.

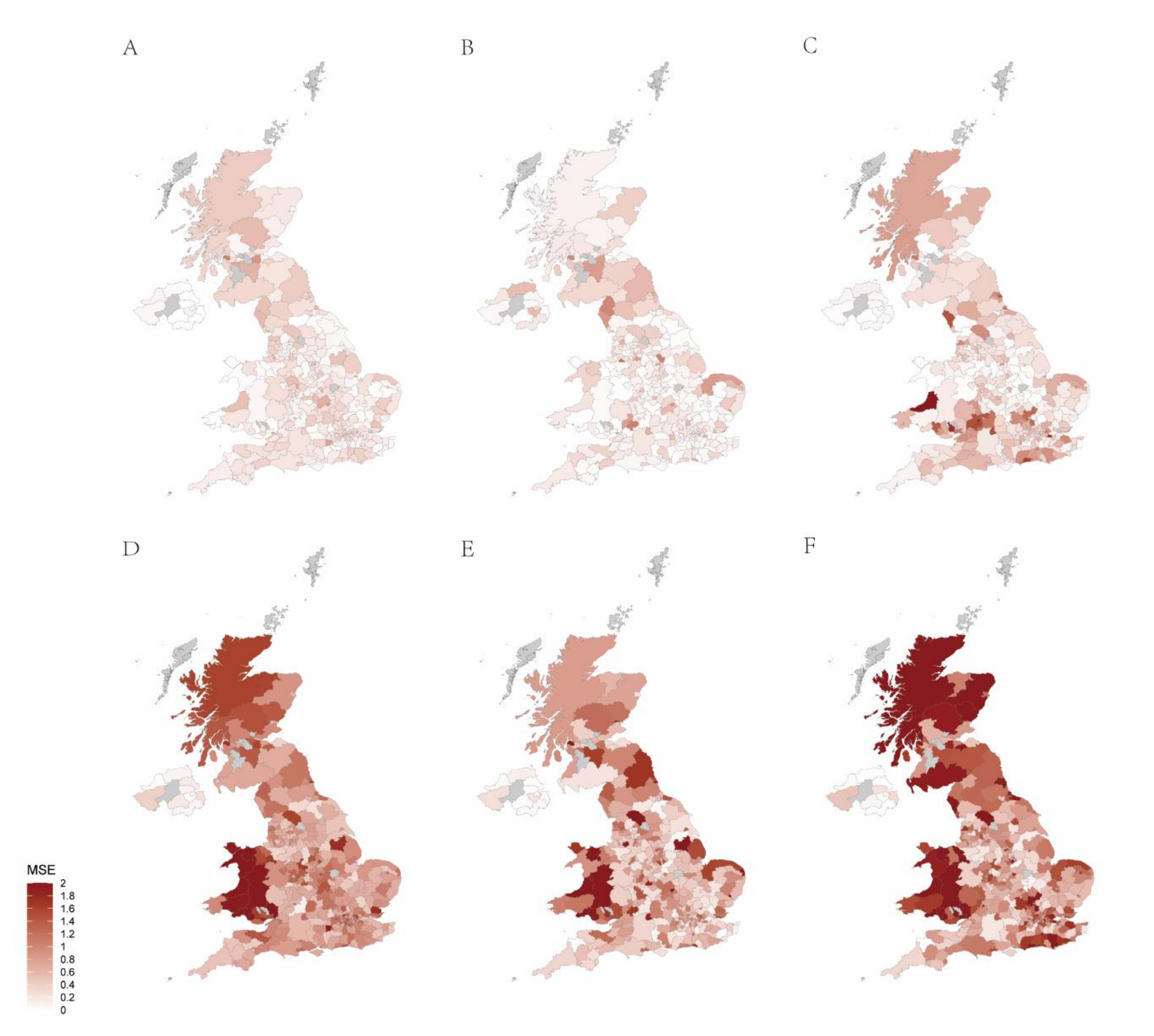

Supplementary Figure 11 Prospective 1-week MSEs by prediction timeframe, week 2/23.

MSE: mean squared error. Panels: 1-week growth rate (A), 2-week growth rate (B) and 3-week growth rate (C) by publication date; 1-week growth rate (D), 2-week growth rate (E) and 3-week growth rate (F) by the collection date of specimen.
