# Supplementary material for: Short-term local predictions of COVID-19 in the United Kingdom using dynamic supervised machine learning algorithms
Source: Commun Med (Lond). 2022 Sep 24;2:119. doi: 10.1038/s43856-022-00184-7 (PMC9509378; doi:10.1038/s43856-022-00184-7)
Supplement: Supplementary file 2 — Description of Additional Supplementary Files [file 43856_2022_184_MOESM2_ESM.pdf]

Description of Additional Supplementary Files

File Name: Supplementary Data 1

Description: Naïve models by prediction and time lag, and the retrospective 4-week MSEs.

File Name: Supplementary Data 2

Description: The retrospective 4-week MSE for different predicator combinations by prediction

File Name: Supplementary Data 3

Description: The 1-week ahead prediction by publication date – the symptoms included in the optimal full models by week.

File Name: Supplementary Data 4

Description: The 2-week ahead prediction by publication date – the symptoms included in the optimal full models by week.

File Name: Supplementary Data 5

Description: The 3-week ahead prediction by publication date – the symptoms included in the optimal full models by week.

File Name: Supplementary Data 6

Description: The underlying data for producing Figure 2.

File Name: Supplementary Data 7

Description: The underlying data for producing Figure 3.

File Name: Supplementary Data 8

Description: The underlying data for producing Figure 4.

File Name: Supplementary Data 9

Description: The underlying data for producing Figure 5.
